# Multiple song features are related to paternal effort in common nightingales

**DOI:** 10.1186/s12862-015-0390-5

**Published:** 2015-06-18

**Authors:** Conny Bartsch, Michael Weiss, Silke Kipper

**Affiliations:** Animal Behavior Group, Freie Universität Berlin, Takustraße 6, 14195 Berlin, Germany; Department of Exposition, Unit Epidemiology, Statistics and Mathematical Modelling, Federal Institute for Risk Assessment (BfR), Max-Dohrn-Straße 8-10, 10589 Berlin, Germany; Chair of Zoology, Technische Universität München, Liesel-Beckmann-Str. 4, 85354 Freising, Germany

**Keywords:** Male quality, Paternal effort, Song complexity, Song sequence, Network analysis, Song category

## Abstract

**Background:**

Sexual ornamentation may be related to the degree of paternal care and the ‘good-parent’ model predicts that male secondary characters honestly advertise paternal investment. In most birds, males are involved in bringing up the young and successful reproduction highly depends on male contribution during breeding. In passerines, male song is indicative of male attributes and for few species it has been shown that song features also signal paternal investment to females. Males of nightingales *Luscinia megarhynchos* are famous for their elaborate singing but so far there is only little knowledge on the role of male song in intersexual communication, and it is unknown whether male song predicts male parenting abilities.

**Results:**

Using RFID technology to record male feeding visits to the nest, we found that nightingale males substantially contribute to chick feeding. Also, we analyzed male nocturnal song with focus on song features that have been shown to signal male quality before. We found that several song features, namely measures of song complexity and song sequencing, were correlated with male feeding rates. Moreover, the combination of these song features had strong predictive power for male contribution to nestling feeding.

**Conclusions:**

Since male nightingales are involved in chick rearing, paternal investment might be a crucial variable for female mate choice in this species. Females may assess future paternal care on the basis of song features identified in our study and thus these features may have evolved to signal direct benefits to females. Additionally we underline the importance of multiple acoustic cues for female mating decisions especially in species with complex song such as the nightingale.

**Electronic supplementary material:**

The online version of this article (doi:10.1186/s12862-015-0390-5) contains supplementary material, which is available to authorized users.

## Background

Parental investment of the two sexes has been proposed as a key promoter of sexual selection in most species of the animal kingdom [1, 2]. In species where both parents are involved in bringing up the young, paternal care is a crucial variable of female choice since females directly benefit from high levels of male investment [3, 4]. Biparental care is especially widespread in birds (~81 % of all species, [5]) with most of the species being socially monogamous [6]. In birds, females have been found to adjust their mating strategies and their own investment according to the expected male participation in offspring rearing [7, 8]. Females may assess paternal qualities on the basis of male secondary traits (‘good-parent’ model [9], but see also [10] and studies cited therein for conflicting results). Mechanisms that have been proposed to explain female assessment of male parenting abilities are similar to ‘good-genes’ or ‘handicap’ models of female choice [11]. These models assume that male extravagant traits come with costs and only males of high phenotypic and/or genetic quality manage to sustain these traits [12, 13]. Traits that have been shown to be related to paternal qualities include morphological characteristics such as plumage coloration [14, 15] or courtship related behaviors [16–19].

In passerines, male song is such a courtship behavior [20] and there is evidence that song serves as predictor for paternal effort. For example, high song rates were found to be associated with higher feeding rates in a few species, and it was suggested that high song rates possibly indicate a male’s foraging efficiency, his ability to defend a high quality territory or individual quality in general [21–23]. Elaborate song flights which are associated with social costs for males advertise future paternal performance in whitethroats *Sylvia communis* [24]. Large repertoires indicate higher feeding rates in male sedge warblers *Acrocephalus schoenobaenus* [25] whereas similar studies in other species failed to find a correlation between measures of song complexity and male care [22, 26–28]. Assuming that song complexity reflects a superior male status [29–31] which has been mostly linked to indirect fitness benefits for females, it might at the same time be an indicator of more direct benefits of female choice (i.e. a male’s ability to provide paternal care). In summary, female songbirds may choose prospective good fathers by paying attention to male song prior to pair formation.

**Fig. 1:**
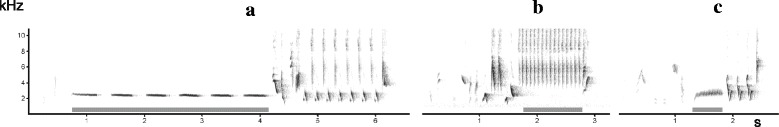
Example songs of nightingale song categories. The acoustic patterns that constitute categories are underlined in grey. (**a**): whistle song, characterized by a homotype series of repeated elements with a narrow frequency band and little or no frequency modulation. (**b**): trill song, characterized by a rapid broad-band trill consisting of repeated elements with little frequency modulation but covering a large frequency range. (**c**): buzz song, characterized by a long, non-repeated buzz element produced by a very fast repetition of sound units in a narrow and rather low frequency range

The common nightingale *Luscinia megarhynchos* is a socially monogamous passerine and from few previous observations it is known that males provide paternal care during breeding. They, for example, feed the female during incubation, provide food to nestlings and readily defend the nest against potential predators [32, 33]; CB, personal observation). Thus, reproductive success seems to be highly dependent on male contribution, making it most probable that in nightingales male parenting abilities are a crucial factor in mate choice. Furthermore, nightingales belong to the most versatile singers of the temperate zone [20]. Males have very large song repertoires of up to 250 different song types (approx. 180 different song types per male, e.g. [34, 35]) with large repertoire birds being older [36], being in better condition and arriving earlier at the breeding grounds [35]. Within repertoires, specific features of certain songs seem to carry information that might be important during different behavioral contexts, which constitutes the basis for the formation of song categories (e.g. buzz songs: [37]; trill songs: [38]; whistle songs: [39, 40]; see also Fig. 1). Furthermore, the nightingale is an excellent model to study the functional aspect of complex ‘syntax-like’ rules of song delivery. Song sequencing in nightingales is neither fully fixed (e.g. singing in an A-Z manner), nor random, but instead follows sequential rules that are partly controlled by early learning and memory retrieval processes, e.g. [41–43]. However, these sequential rules are not fully deterministic, but offer potential for behavioral plasticity both short- and long-term. For example, males change their repertoire composition and song sequencing with age, which is most probably attributed to an adjustment to the breeding population [44–46], or they change the sequencing of songs in response to playbacks [47]. Also, the usage of specific song types (i.e. song categories) is related to different behavioral contexts or different times during the day and the breeding cycle, e.g. [39, 48–52] which additionally affects the sequencing of songs. Very recently it has been shown that the sequential ordering of song types is related to male attributes. For example, older males sing their songs more ordered (i.e. they sing song sequences repeatedly in the same order) and membership to a population seems to be encoded in song sequencing (i.e. members of the same population share song sequences) [46, 47]. These findings imply that the sequential ordering of nightingale song may carry information which is relevant for females during mate choice.

If a male’s song is also related to his parenting qualities, and if females use song to choose ‘good fathers’ has not been investigated in nightingales so far. To date, there are neither studies systematically investigating paternal care in the nightingale, nor studies looking at the relationship between song and parenting qualities. Here, we investigated the potential function of male song as an indicator for future male parental care. To do so, we analyzed paternal care by continuous recording of male provisioning behavior at the nest across several days. Also, we analyzed male nocturnal song with special focus on repertoire size, repertoire composition, and the sequencing of songs as possible candidates for the advertising function of male song. We hypothesized that males who are more committed to paternal care are ‘better’ singers.

## Results

### Male feeding effort

**Fig. 2:**
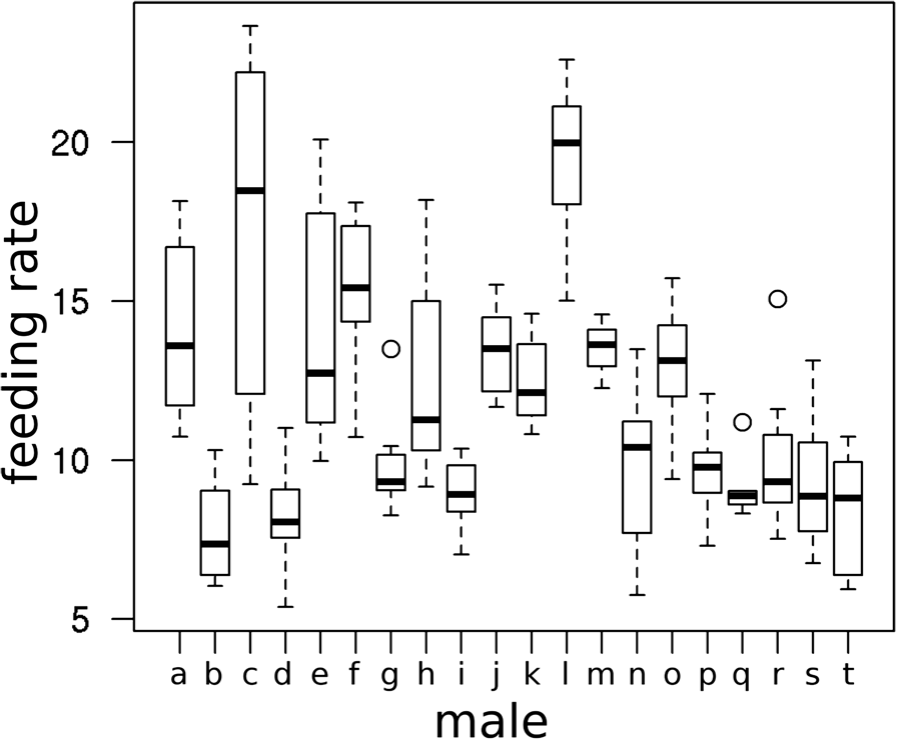
Male variability in feeding behavior. Shown are individual feeding rates of all males (*n* = 20) during the recording period. Variability between males is higher than within males. For statistics see text

The analysis of video recordings of four nests during the late nestling phase (chicks older than eight days) confirmed that male nightingales contribute to chick feeding. A comparison of the relative effort of both parents showed that males and females on average contributed equally to feeding, whereby individuals showed high variance in their feeding effort (rate [nest visits/h] males: 15.9 ± 4.9 (mean ± SD); rate [nest visits/h] females: 15.5 ± 20.0 (mean ± SD); for details see Additional file 1: Table S1). For another 20 males, we calculated feeding rates from RFID recordings during morning and evening hours of days 5 to 12 of chick age. The feeding rate across all males was 11.4 ± 3.8 (mean ± SD), i.e. males on average visited the nest eleven times per hour (note that the difference in feeding rate observed in the pilot study is most probably attributed to an increase of feeding activity with nestling age (see Additional file 1: Figure S3)). Males varied considerably in their feeding behavior, whereas feeding rates were stable within males across the recording period (ANOVA, *n* = 20, *F* = 12.46, *P* < 0.001; Fig. 2).

### Relationship between male song and feeding behavior

**Fig. 3:**
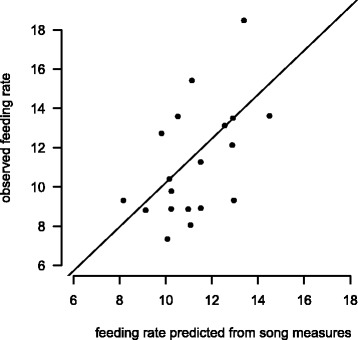
Relation between actual and predicted male feeding rates. Using the support vector machine algorithm we were able to reliably predict male feeding rates on the basis of the combination of four different song features (average shortest path length, buzz, whistle and trill repertoire size). This is indicated by the strong correlation of the actual feeding rates observed in the field and the rates predicted by the algorithm (calculation based on 20 males). For statistics see text

First, we identified those variables with the strongest relation to male feeding rates. The LASSO algorithm resulted in four song measures: average shortest path length, buzz repertoire size, whistle repertoire size and trill repertoire size (with regression coefficients in decreasing order: 0.75, 0.46, 0.14 and 0.005). The four song measures were all positively correlated with male feeding rates (Spearman rank correlation, *n* = 20, all *P* < 0.05; see Additional file 1 for details on statistics and Additional file 1: Figures S4-S7). Thus, males with a more ordered singing style (i.e. male who repeatedly sang the same sequential order of song types indicated by higher average shortest path values) and more complex song repertoires (indicated by larger buzz, whistle and trill repertoire sizes) fed their chicks more often. The effects of all other song variables were reduced to zero by the LASSO algorithm. Besides song, the ‘number of hatchlings’ affected male feeding rates (regression coefficient: 0.43) whereas ‘year’ did not have an influence. With this subset of variables (shortest path length, buzz repertoire size, whistle repertoire size, trill repertoire size and number of hatchlings) we trained a support vector machine algorithm to test the combinatory predictive power of the variables. We found that the performance of the support vector machine was very good since the predicted feeding rates by the algorithm were highly correlated with the actual feeding rates observed in the field (Spearman rank correlation, *n* = 20, *r* = 0.54, *P* = 0.014; see Fig. 3). Thus, feeding rates of males can be reliably predicted based on four combined song measures plus number of hatchlings.

## Discussion

Our study confirmed previous observations that male nightingales considerably contribute to chick feeding with large differences between individual males. These differences in male feeding effort were related to several features of male nocturnal song. When analyzing which song features reliably predict male parenting abilities we found that male feeding effort can be assessed on the basis of the combination of four song measures, namely song orderliness, buzz, whistle and trill repertoire size. More specifically, males that sang their song sequences more ordered and sang many different buzz, whistle as well as trill song types contributed more to chick feeding.

Our study provides the first thorough quantification of male feeding effort in nightingales including exemplary results on relative feeding contributions of both parents. Our results on male feeding behavior fit well the described pattern that in socially monogamous and monomorphic species male parental care is common, whereas reduced male care, or its absence, is often correlated with polygyny and with sexual dimorphism in birds [6, 53]. Most interestingly, we found that the extent of future paternal effort is advertised by male nocturnal song prior to pair formation. Evidence from the literature for this specific indicator function of song is still rare, especially for species with elaborate song (to compare: [25]). Also, we identified song characteristics that have not been described before to be related to paternal qualities in birds.

To the best of our knowledge, this is the first study reporting a relationship between sequential ordering of songs (*here* song orderliness) and paternal care in birds. Although the sequential organization of birdsong has been intensively studied ([54] and studies cited therein), there is only very scarce knowledge on the biological and adaptive value of song sequencing, e.g. [47, 55, 56]. In our study, the orderliness of male song sequencing indicated increased paternal investment. Given that song orderliness is also associated with male age [47], females choosing an orderly singing male might benefit from a male who signals viability and experience with the breeding grounds (factors significantly enhancing reproductive success in birds [57, 58]) *and* his willingness to care for the offspring. Together these findings support the idea that the sequential ordering of birdsong carries relevant information during communication which may be directed to female listeners and thus, may have evolved by female choice [59].

In addition to song sequencing, measures of song complexity (*here* buzz, whistle and trill song repertoire size) were correlated with higher feeding rates in our study. A similar relationship between song complexity and male provisioning has so far only been reported for the sedge warbler [25]. In other studies, the value of mating with large repertoire singers has mostly been linked to indirect (i.e. genetic) benefits for females [29, 60], or to other aspects of male quality (e.g. territory quality: [61, 62]; body and health condition: [63, 64]; early condition: [30, 65]; age: [66]). For nightingales, the role of repertoire composition and size in interspecific contexts is not yet fully understood since we for example, lack evidence that females are attracted by more complex song. On the other hand, there is correlative data on the potential indicator function of large repertoires [35, 36] and playback studies showed that females prefer songs containing many different buzz or whistle songs [37, 40]. Since in our study the number of different buzz, whistle and trill song types was highly correlated with repertoire size, it might be that females use the variety within song categories as a proxy for overall song complexity (i.e. repertoire size). Alternatively or additionally, the fine structure of either buzz, whistle and trill elements might encode information on male quality or motivation, and thus be relevant for females [67, 68]. To date, there is no direct evidence that the production of these elements is physically challenging for male nightingales. However, it was shown that males differ in the performance of these song structures, and that performance differences are related to male traits reflecting quality [37, 40, 69]. Whether such performance measures predict paternal investment has not yet been investigated in songbirds, but there is accumulating evidence that even in large repertoire species the performance of fine-scale song structures is of adaptive value [70–72]. Although we cannot say what exact song features female nightingales finally use to assess males, it seems most likely that females use both, the quantity (i.e. repertoires) *and* quality (i.e. performance) of songs to assess a male’s adequacy as a mating partner.

In socially monogamous taxa there are different models on the extent of paternal investment depending on self-attractiveness [73–75]. Although our data do not allow evaluating relative parental feeding effort in more detail (for example whether male attractiveness affected maternal effort, [76]) we found that better singers fed their offspring more often. This supports the ‘good parent’ hypothesis which predicts that an individual’s attractiveness should reliably and positively indicate parental effort [9]. The quantification of further measures in the feeding context (such as for example female feeding contributions and chick mass or chick growth) will allow to draw a more detailed picture on the complex relationships between singing and parental investment in nightingales. With the method applied (support vector machine algorithm) we were able to identify four song parameters that gave strong predictive power for male feeding effort. This strategy (combinatory usage of different song features) might be also highly adaptive for females especially when the evaluation of single parameters might be time consuming (e.g. assessment of full repertoires), or is hindered or not possible (e.g. song structures especially prone to attenuation and degradation). The integration of multiple (vocal) cues might therefore enhance accurate and fast assessment of males and may thus be a prerequisite for successful reproduction [77, 78]. This seems particularly feasible in species that have evolved complex vocal signal systems such as the nightingale where signals may contain multiple messages for different receivers [79].

## Conclusions

We conclude that different aspects of male nightingale song honestly advertise direct fitness benefits to females. Here, measures of song complexity and song sequencing reliably predict paternal investment and thus might be important for female mating decisions. We suggest that, during mate choice, female nightingales rely on multiple song cues to quickly gather information about signaller quality.

## Methods

### Study site and subjects

The study was carried out on a population of nightingales in the Golmer Luch, Potsdam, Germany (52.4°, 12.97°). Since 2009, males have been monitored, banded and recorded in this area (see [40, 80] for details on breeding site and data acquisition). For the present study, subjects were 20 resident males (identifiable by unique color ring combinations) with established territories between 2010 and 2012 (each male was only sampled once). Males were regularly observed in their territories to confirm male identity and male pairing status throughout the breeding season. Nocturnal singing was recorded early in the breeding season when males were most probably still unpaired since males cease nocturnal song after pair formation [81]. Nests were located by observing nest related activities such as nest building, emission of alarm calls or feeding flights. We documented chick development (chick measurements and photos) within nests to estimate chick age ± one day by comparing chick development to reference nests with known hatching date.

### Song analyses

High quality recordings of nocturnal song (1130 pm–0300 am) were used for analyses. Song was recorded in 3–10 m distance of a singing male with a Sennheiser ME66/K6 directional microphone which was connected to a portable Marantz PMD-660 Compact Digital Recorder. All sound analyses were conducted with the software Avisoft SASlab Pro 4.52 (R. Specht, Berlin, Germany). Recordings were down-sampled to 22.05 kHz, high pass filtered (0.8 kHz, Butterworth) and amplitude normalized to 75 %. First, we determined repertoire sizes of all males via visual inspection of 533 consecutive songs, equalling approximately 1 h of singing, which has been proven to result in saturated repertoire curves (for details see [34, 35, 44]. Since nightingale song types have been assigned to song categories (e.g. buzz songs [37], trill songs [38] and whistle songs [39]), we further analyzed repertoire composition by determining how many different types occurred within each category (i.e. determination of whistle, trill and buzz song repertoire) and how often songs of these categories were sung (i.e. whistle, trill and buzz song occurrence irrespective of type, see also Table 1 and Fig. 1 for definitions and song examples). We also determined the degree of song orderliness using network analysis, where song sequences are translated into networks of song types as nodes with song transitions as connectors (following [47]). We calculated two network measures that have been shown to be particularly well suited to describe the sequential order of nightingale singing (i.e. with large repertoires and complex rules of song retrieval): average shortest path length and transitivity (for details on procedure see [47]). Average shortest path length is sensitive to long linear transitions of songs and transitivity refers to the interconnectivity of songs in sequential proximity [47] (Table 1).

**Table 1:** Overview on nightingale song measures

Measure	Definition	Mean ± SD	CV
Repertoire size	Number of different song types	181 ± 35	0.19
Whistle repertoire size	Number of whistle song types in the repertoire	28 ± 5	0.18
Whistle song occurrence	Number of whistle songs (independent of types) in the song sample	74 ± 17	0.23
Trill repertoire size	Number of trill song types in the repertoire	20 ± 4	0.2
Trill song occurrence	Number of trill songs in the song sample	64 ± 10	0.15
Buzz repertoire size	Number of buzz song types in the repertoire	5 ± 1	0.33
Buzz song occurrence	Number of buzz songs in the song sample	14 ± 4	0.3
Shortest path	Network measure: the path connecting two nodes with the minimal number of nodes in between	4.66 ± 0.84	0.18
Transitivity	Network measure: a measure for the probability that adjacent nodes of a given node are connected as well	0.18 ± 0.03	0.17

Name, description, and mean values for song features under study. All measures refer to song samples of 533 consecutive songs. Mean values are shown for 20 males. Both network measures were calculated and then averaged for all possible nodes or pairs of nodes in a network

### Male feeding

In a pilot study on nestling provisioning in nightingales (conducted in 2008 and 2009), we obtained video recordings (recording time: ~2 h per nest) from four nests during the late nestling phase (chicks older than eight days). Videos were analyzed to describe male feeding effort with special focus on the relative contribution of both parents to chick feeding. In all nests, male and female birds were distinguishable by colored leg rings.

Between 2010 and 2012 we used radio frequency identification (RFID) technology to record male visits to the nest (Assion Electronic, Niederkassel, Germany). To do so, a passive micro transponder (Ø 2.2 mm × 12 mm < 0.1 g) was glued to one of the plastic rings which were fitted to a male’s leg during bird banding. The RFID device consisted of a data-logging device (LID-650 decoder, Assion Electronic) which was connected to a circular antenna (Ø 120 mm) and powered by an energy supply module (Assion Electronic). The antenna was positioned around the upper rim of the nest and the other equipment was placed on the ground nearby and covered with plant material not to distract the birds or attract the attention of predators. When a focal male approached the antenna (<10 cm), arrivals and departures were registered and saved exact to the second. From this we calculated male visiting rate (number of visits/h). Since this method provided data on male visits to the nest, but not on actual provisioning, we compared the RFID data with data from a simultaneous video recording for one exemplary nest to validate the accuracy of the automated monitoring system and the relevance of the measure obtained. We found that RFID data correlated well with male nest attendance in the video recording and that the male brought food whenever he approached the nest (comparison of 17 nest visits in ~80 min). Thus, we concluded that male visiting rate was a good proxy for male feeding rate.

The RFID system collected data on male feeding behavior continuously from dusk till dawn registering all male nest visits during this time for several days between days 5 to 12 of chick age (note that not all males were sampled during the same nestling stages and males differed in total recording time; for more information and original data on male feeding efforts across daytimes and several nestling stages see Additional file 1: Figure S1–S3). For the analyses on the relationships between male feeding behavior and male song we only used data from the morning (feeding rate from 1^st^ nest visit in the morning until 10 am) and evening (feeding rate from 6 pm until last nest visit in the evening) hours since these might be the biologically most critical periods due to feeding breaks during night. As a composite measure of feeding effort per male, we calculated the median of these rates for each male.

### Statistics

All data were analyzed using R (R Development Core Team, v. 3.1.1; [82]) and statistical significance was set at *P* ≤ 0.05 (two-tailed).

To investigate the variability of feeding rates among males we calculated an analysis of variance (ANOVA). Next, we were interested in the identification of candidate song features that might be useful in predicting later parental investment of males. In a first step of analysis we reduced the number of song variables potentially related to male feeding rate by using the least absolute shrinkage and selection operator (LASSO) as implemented in the R package ‘glmnet’ [83]. We included feeding rate as response measure and the nine song measures (see Table 1) as fixed factors. Since it has been shown that feeding rates vary with brood size (e.g. [84, 85]) and might change between years (e.g. [86, 87]), we included ‘number of hatchlings’ and ‘year of study’ as further factors to account for possible effects of confounding variables. Next, we investigated if the song features and other variables identified by the LASSO method can be used to predict male feeding rates. To do so, we used all variables of the reduced data set to train a support vector machine algorithm as implemented in the R package ‘e1071’ [88]. The training was performed as follows: we excluded one individual and trained the support vector machine with the measures of the remaining 19 males and predicted a feeding rate for the excluded individual from this data. This procedure was repeated until each of the 20 males was excluded and his feeding rate was predicted. To finally estimate the performance of the support vector machine we compared the predicted feeding rates with the actually observed feeding rates by calculating a Spearman rank correlation. To investigate the influence of each single song variable of the reduced data set in more detail, we additionally calculated Spearman correlations between song variables and male feeding rate (see also Additional file 1 for detailed results).

## Additional file

Additional file 1:
**Supplementary data.** The supplementary data include more detailed information on methods and results of song analysis and feeding behavior.

## Abbreviations

RFID: Radio frequency identification
SD: Standard deviation
