# Supplementary material for: Multiple song features are related to paternal effort in common nightingales
Source: BMC Evol Biol. 2015 Jun 18;15:115. doi: 10.1186/s12862-015-0390-5 (PMC4471916; doi:10.1186/s12862-015-0390-5)
Supplement: Additional file 1: — Supplementary data. The supplementary data include more detailed information on methods and results of song analysis and feeding behavior. [file 12862_2015_390_MOESM1_ESM.pdf]

Supplementary data

Song Analysis

We analyzed 9 song parameters of male nocturnal song. Several of these song features were correlated: Buzz repertoire size, whistle repertoire size and trill repertoire size were all positively correlated with overall repertoire size (Pearson correlation, buzzrep: r = 0.65, P = 0.002; whisrep: r = 0.65, P = 0.0019; trillrep: r = 0.88, $P \leq 0.001$, all n = 20) whereas the occurrence of song types within each category did not correlate with category repertoire size (Pearson correlation, all $P > 0.05$). Furthermore, average shortest path length was highly correlated with repertoire size (Spearman rank correlation, r = 0.88, $P \leq 0.001$, n = 20) and transitivity showed an almost significant (negative) correlation with repertoire size (Spearman rank correlation, r = -0.43, P = 0.056, n = 20). All other song features were not related to each other.

Feeding behavior

a) *Video recordings*

We conducted a pilot study on male and female feeding behavior during the late nestling phase (chicks older than 8 days) in 2008 and 2009. Four nests (different from nests that were monitored by the RFID system) were video recorded for about 2 hours. Male and female birds were distinguishable by colored leg bands. Results are summarized in table 2.

Table 1: Male and female feeding behavior during the late nestling phase, n = 4 nests.

Year	♂ age*	Rec time [min]	No. visits both parents	No. of visits with identified sex	No. of ♂ visits	No. of ♀ visits	[%] ♂ feeding	[%] ♀ feeding	feeding rate [per hour] both parents	♂ feeding rate	♀ feeding rate
2009	2	104	65	56	33	23	59	41	37,5	19,0	13,3
2009	2	110	27	24	24	0	100	0	14,7	13,1	0,0
2008	2	200	58	50	35	15	70	30	17,4	10,5	4,5
2008	2	92	100	100	32	68	32	68	65,2	20,9	44,3
				Mean	*31,0*	*26,5*	*65,2*	*34,8*	*33,7*	*15,9*	*15,5*
				SD	*4,8*	*29,3*	*28,1*	*28,1*	*23,3*	*4,9*	*20,0*

*male age in nightingales being determined as 'yearling' or '*at least* two years old' (Mundry and Sommer 2007)

b) *RFID data*

Between 2010 and 2012 we collected data on male feeding behavior (n = 20) via an automated electronic monitoring system (RFID) during different stages of the nestling phase. Male feeding behavior was recorded between days 5 to 12 of chick age whereby individual males were recorded at different days: for example, only single males were recorded at day 5, 11 and 12 of chick age, whereas most males where recorded between 7 and 9 days of chick age (see also table 3). For data exploration, we assigned recordings to different times of the day: morning (1st visit in early morning – 1000 am), noon (1000 am – 0200 pm), afternoon (0200 pm – 0600 pm) and evening (0600 pm – last nest visit late evening). This allowed us to estimate the variability of male feeding behavior across the nestling phase and over the day since it has been shown that feeding rates can vary during different recording periods (Rose 2009, Mariette et al. 2011).

Table 2: Number of males that have been RFID-recorded during different nest stages (as shown in days of chick age) and during different times of the day (morning, noon, afternoon and evening).

	Chick age in days							
Day time	5	6	7	8	9	10	11	12
morning (1st visit-10am)	0	2	15	18	18	15	2	2
noon (10am-2pm)	1	7	17	17	17	12	2	2
afternoon (2-6pm)	1	12	19	18	16	5	2	2
evening (6pm-last visit)	2	14	18	19	14	4	2	1

From the RFID data we extracted several parameters of male nest visiting behavior. We calculated the duration of stay at the nest [s], the time interval between nest visits [min] and male visiting rate [number of visits/hour]. Results are depicted in figures 1-3.

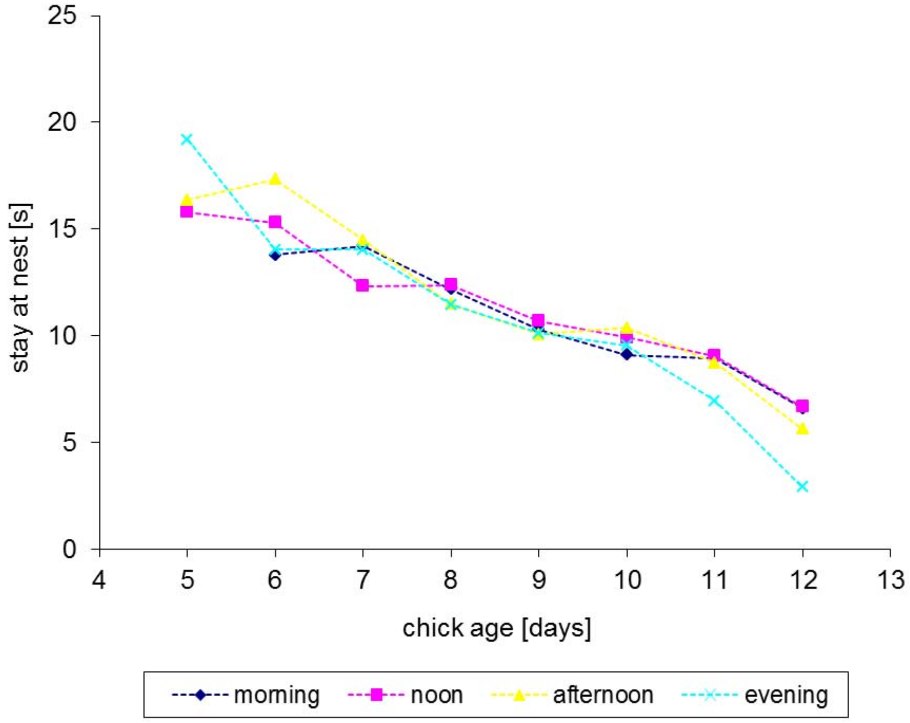

Figure 1: Duration of male stay at the nest in seconds during different times of the day across chick age. Shown are means per recording period whereby the number of males recorded differs between single data points (see table 2).

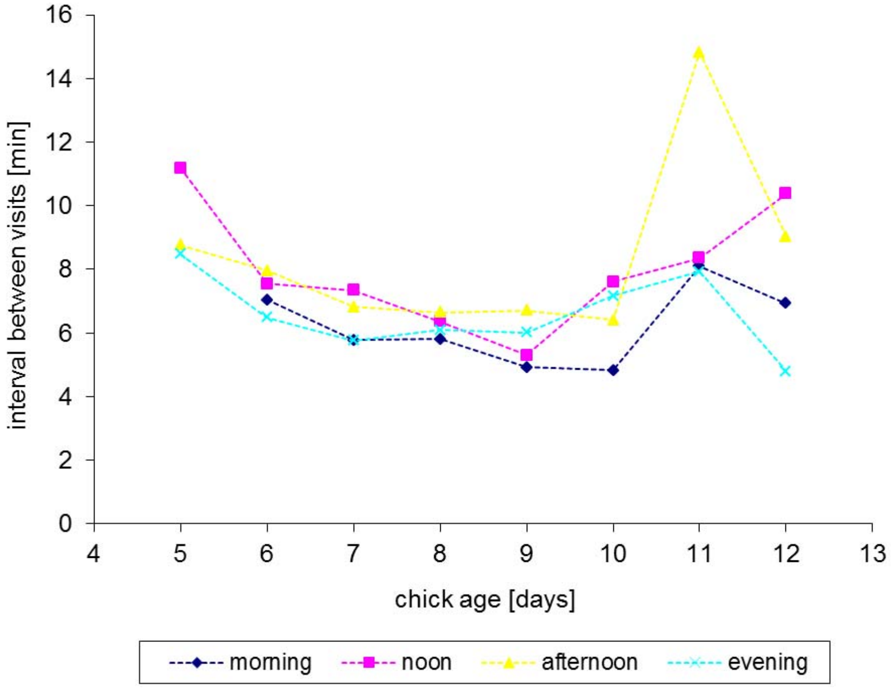

Figure 2: Interval between visits in minutes during different times of the day across chick age. Shown are means per recording period whereby the number of males recorded differs between single data points (see table 2).

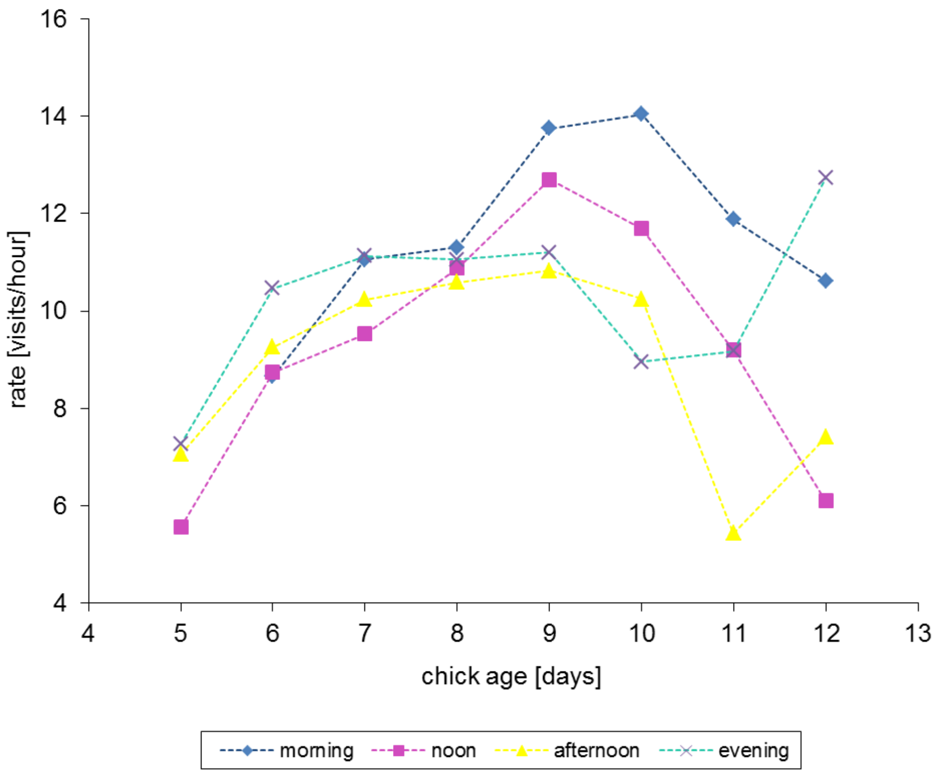

Figure 3: Male nest visiting rate during different times of the day across chick age. Shown are means per recording period whereby the number of males recorded differs between single data points (see table 2).

Relationship between male song and feeding behavior

To investigate the influence of each single song variable of the reduced data set (song features with the strongest relations to m ale feeding identified by the LASSO algorithm) in more detail we additionally calculated Spearman correlations between song variables and male feeding rate. Here, average shortest path was positively correlated with male feeding rate (Spearman's rank correlation, n = 20, r = 0.72, P = 0.0003; see figure 4). Since higher average shortest path values are related to a more ordered singing style where males repeatedly sing the same sequential order of song types, the correlation indicates higher feeding rates in males with more ordered song. Buzz, whistle and trill repertoires were all positively correlated with

male feeding rates (Spearman's rank correlation, buzz repertoire: r = 0.43, P = 0.06; whistle repertoire: r = 0.50, P = 0.02; trill repertoire: r = 0.60, P = 0.005; all n = 20; see figure 5, 6 and 7) indicating higher feeding effort in males with more complex song.

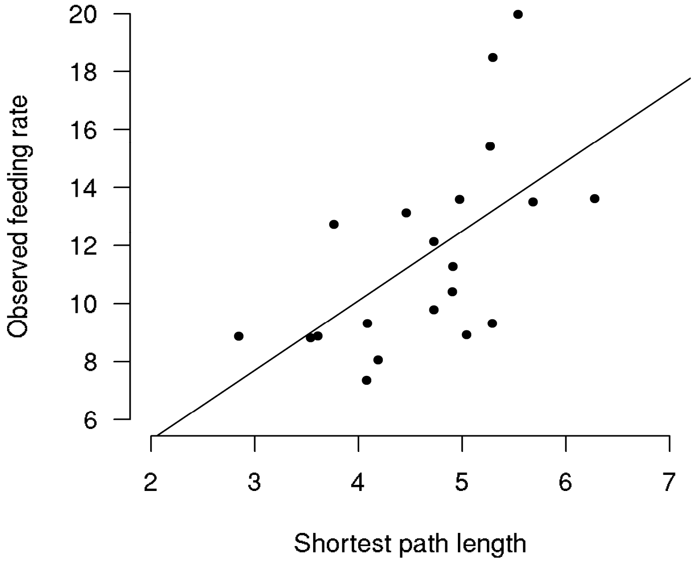

Figure 4: Relation between male feeding rate (median from morning and evening feeding rates across recording period) and average shortest path length, n = 20 (see text for statistics).

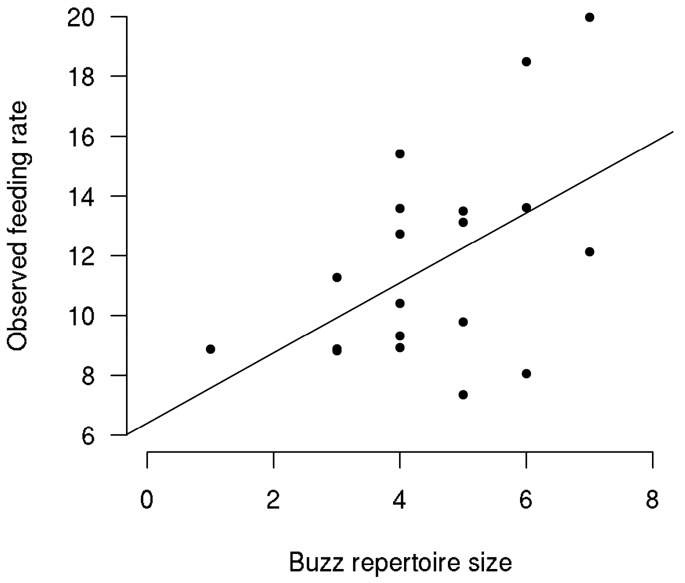

Figure 5: Relation between male feeding rate (median from morning and evening feeding rates across recording period) and buzz repertoire size, n = 20 (see text for statistics).

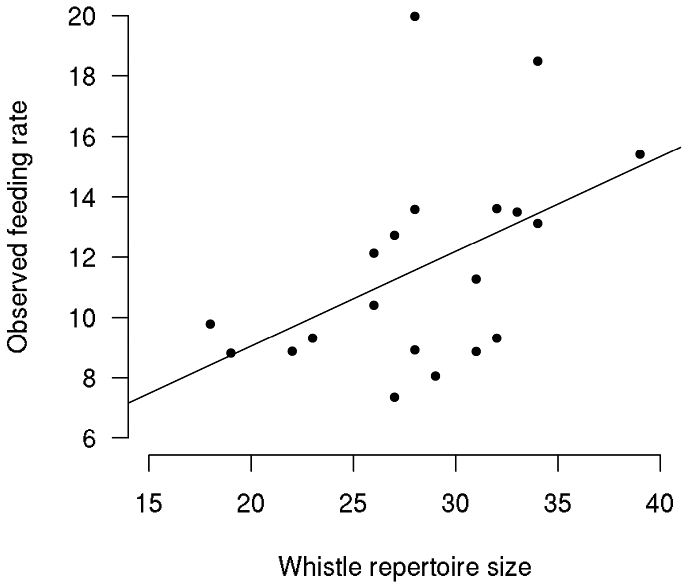

Figure 6: Relation between male feeding rate (median from morning and evening feeding rates across recording period) and whistle repertoire size, n = 20 (see text for statistics).

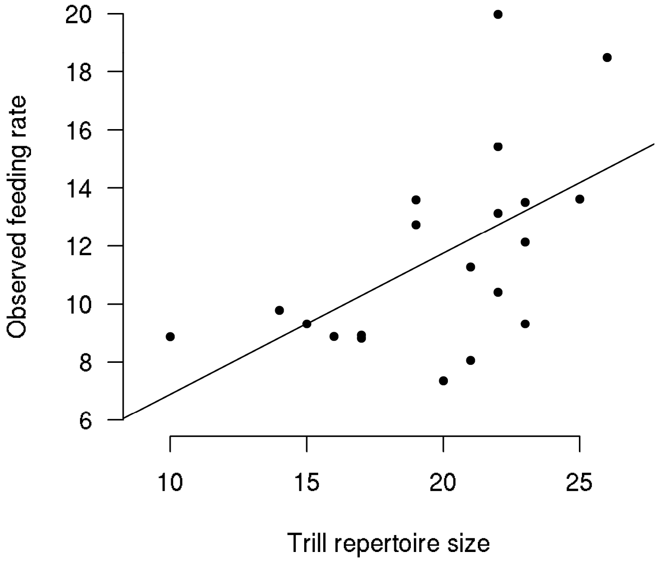

Figure 7: Relation between male feeding rate (median from morning and evening feeding rates across recording period) and trill repertoire size, n = 20 (see text for statistics).

References

Mariette MM, Pariser EC, Gilby AJ, Magrath MJ, Pryke SR, Griffith SC: **Using an electronic monitoring system to link offspring provisioning and foraging behavior of a wild passerine.** *The Auk* 2011, **128**:26-35.

Mundry R, Sommer C: **Ein neues Gefiedermerkmal zur Altersbestimmung bei Nachtigallen (*Luscinia megarhynchos*).** *Limicola* 2007, **21**:131-139.

Rose AP: **Temporal and individual variation in offspring provisioning by Tree Swallows: A new method of automated nest attendance monitoring.** *PloS ONE* 2009, **4**:e4111.
